# A meta-study of qualitative research examining determinants of children’s independent active free play

**DOI:** 10.1186/s12966-015-0165-9

**Published:** 2015-01-24

**Authors:** Homan Lee, Katherine A Tamminen, Alexander M Clark, Linda Slater, John C Spence, Nicholas L Holt

**Affiliations:** Faculty of Physical Education and Recreation, University of Alberta, Edmonton, Alberta Canada; Faculty of Kinesiology and Physical Education, University of Toronto, Toronto, Ontario Canada; Faculty of Nursing, University of Alberta, Edmonton, Alberta, Canada; University of Alberta Library, University of Alberta, Edmonton, Alberta Canada

**Keywords:** Meta-synthesis, Safety, Parents, Theory

## Abstract

**Purpose:**

To produce a meta-study by completing a systematic review of qualitative research examining determinants of independent active free play in children.

**Method:**

Following systematic electronic and manual searches and application of inclusion/exclusion criteria, 46 studies were retained and subjected to meta-method, meta-theory, and meta-data analyses, followed by a final meta-synthesis.

**Results:**

Identified determinants of independent active free play were child characteristics (age, competence, and gender), parental restrictions (safety concerns and surveillance), neighborhood and physical environment (fewer children to play with, differences in preferences for play spaces between parents and children, accessibility and proximity, and maintenance), societal changes (reduced sense of community, good parenting ideal, changing roles of parents, privatization of playtime and play spaces), and policy issues (need to give children voice). An ecological model depicting these factors, and the relationships therein, was created.

**Conclusions:**

This comprehensive meta-study helps establish a knowledge base for children’s independent active free play research by synthesizing a previously fragmented set of studies. Parents’ perceived safety concerns are the primary barrier to children’s active free play. These safety concerns are moderated by child-level factors (age, competence, gender) and broader social issues. Interventions should focus on community-level solutions that include children’s perspectives. From a methods perspective, the reviewed studies used a range of data collection techniques, but methodological details were often inadequately reported. The theoretical sophistication of research in this area could be improved. To this end, the synthesis reported in this study provides a framework for guiding future research.

**Electronic supplementary material:**

The online version of this article (doi:10.1186/s12966-015-0165-9) contains supplementary material, which is available to authorized users.

## Background

Regular physical activity is associated with improved health status, primary and secondary prevention of chronic diseases, reduced risk of premature death, improved cognitive functioning, academic achievement, and lower depression [1-6]. The more physical activity, the greater the health benefits [7]. Yet, in many developed countries children do not engage in sufficient physical activity (e.g., [8]).

Findings from systematic reviews consistently demonstrate the time children spend outdoors correlates positively with their physical activity [9-12]. Play is one of the main ways in which children engage in physical activity outdoors. In the current study we defined play as child-organized and initiated spontaneous and voluntary activities that take place outdoors, outside of school hours and organized/adult-directed settings, and clearly require children to engage in physical activity [13,14]. We termed this type of play independent active free play. Mirroring patterns of physical activity participation, children’s independent active free play has declined significantly over the past 50 years [15-17]. Consequently, researchers, policy-makers, and practitioners have realized the need to address factors that influence children’s engagement in independent active free play. A systematic review of the literature will help to establish a knowledge base to inform future attempts to study and promote independent active free play.

The current study was intended to advance the literature beyond existing reviews. For example, Carver, Timperio, and Crawford [18] completed a narrative review of (qualitative and quantitative) studies that examined associations between neighborhood safety and physical activity among youth. However, this was not a systematic review and it focused on stranger danger and road safety, thereby excluding other determinants of children’s independent active free play. Allender, Cowburn, and Foster [19] reviewed 24 qualitative studies focused broadly on “sport and physical activity” (p. 826) but only included studies of participants who lived in the UK. McCormack Rock, Toohey, and Hignell [20] reviewed 21 qualitative studies published from 1995 to 2008, which focused on characteristics of urban parks only. In contrast, the current study was not limited by a focus on particular determinants of independent active free play, geographical area, urban locale, or timeframe in order to provide an up-to-date and comprehensive analysis and synthesis of the literature.

We systematically reviewed only *qualitative* studies of children’s independent active free play. Qualitative research can add to knowledge derived from quantitative studies. For instance, two systematic reviews of quantitative studies found proximity of parks/playgrounds and access to facilities/programs had a consistently positive relationship with physical activity [12,21], whereas two others found no association [10,22]. McCormack et al.’s [20] review of qualitative research demonstrated park users’ perceptions of attributes including safety, aesthetics, amenities, in addition to proximity, were important for encouraging park use. These authors concluded that individuals’ perceptions of the social environment “entwine inextricably with perceptions of the physical environment” (p. 712). In this case, qualitative findings describing park users’ perceptions of park use help explain why there are inconsistent findings from objectively measured studies (i.e., users’ perceptions of quality of parks influence their use).

There is a substantial body of qualitative literature examining independent active free play. These qualitative studies have been produced by researchers from a range of academic disciplines such as geography, health promotion, physical activity, obesity, developmental psychology, recreation and leisure, and city planning. Individual qualitative studies can inadvertently produce a somewhat fragmented body of knowledge, especially in the case of studies conducted across numerous disciplines. A systematic review of qualitative studies can synthesize findings and advance the knowledge base about a phenomenon [23,24]. Synthesizing the fragmented literature will provide a useful resource for guiding future research on this topic across disciplines.

Given the limitations of previous reviews [18-20], there remains a need to systematically review qualitative studies of independent active free play. The purpose of this study was to produce a meta-study by completing a systematic review of qualitative research examining determinants of independent active free play in children. The goals of qualitative meta-studies are to produce new and integrative interpretations of findings that are more substantive than those resulting from individual studies alone [25].

## Methods

### Procedure

An information specialist with expertise in advanced database searching, in consultation with two other researchers, created a search strategy that combined ‘active free play’ terms with a filter designed to limit results to qualitative studies (see Additional file 1 for examples). Several databases were searched (including EBSCO Sport Discus, Academic Search Complete, Child Development & Adolescent Studies, Ovid MEDLINE(R), Ovid ERIC, Ovid PsychInfo, Ovid EMBASE, ProQuest Sociological Abstracts, Web of Science Core Collection, and Scopus) from inception to ‘present’ with the final search completed on December 1^st^ 2013. Weekly Google Scholar searches along with manual searches of reference lists of obtained studies and authors’ websites were also completed. Finally, after the manuscript was subjected to peer review, an updated search was completed on August 16^th^ 2014, which resulted in the addition of a further 4 studies.

### Inclusion/exclusion criteria

To be considered for inclusion, studies must have met the following criteria. First, they examined issues associated with children’s independent active free play, defined as child-organized and initiated spontaneous and voluntary activities that take place outdoors, outside of school hours and organized/adult-directed settings, and clearly require children to engage in physical activity [13,14]. Studies of sedentary forms of play (e.g., symbolic, imaginative, video games) and adult-directed activities (e.g., organized sport, adult-directed play during and after-school) were excluded. Second, they must have been original research published in peer-reviewed academic journals. Literature reviews, methodological papers, conceptual/theoretical papers, and government, non-governmental organization, and non-profit organization reports were excluded. Finally, studies were included if they reported primary data using at least one qualitative research method (e.g., interview, observation). Mixed methods studies were included if qualitative data could be separated and examined independently from quantitative data. Studies that included open-ended survey questions were included if raw data (i.e., quotes) were reported.

### Screening and selection of studies

**Figure 1:**
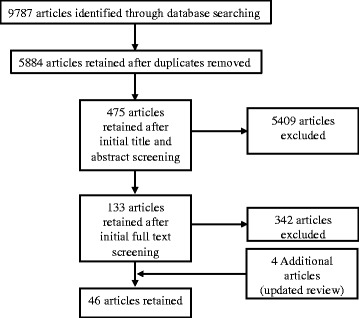
**Manuscript retrieval.**

The initial computerized searches produced 5,884 studies after removal of duplicates (Figure 1). One co-author and three research assistants completed initial screening of abstracts and titles under the supervision of the team leader. At least two people screened every abstract/title, and all met on a weekly basis to discuss any issues and ensure a consistent approach to screening studies. Only studies that did not meet the initial sampling criteria in some clear and incontestable manner were excluded at this stage to minimize the risk that relevant studies were discarded [23]. Following initial screening 457 studies were retained.

Working in teams of two, under the supervision of the team leader, full texts of each of the 457 studies were reviewed. Following the application of the inclusion/exclusion criteria, 133 studies were retained after full text screening. However, the range of studies returned was too diverse for meaningful comparison, so further screening and selection was required [26]. For instance, upon further screening we identified studies that initially appeared to examine independent active free play but focused exclusively on activities occurring during school recesses, supervised/adult-directed after-school periods, and kindergarten programs [27,28]. These studies in adult-directed settings^a^ were excluded because the strong element of adult involvement meant that they were not studies of independent active free play as defined in this study. For instance, key findings from some of these studies emphasized the role of the teacher in constraining play opportunities in the schoolyard, such as strictly controlling when and where children were allowed to play and their movement in and around school yards [28].

Second, the original searches returned qualitative studies of outdoor *physical activity in general* that were not sufficiently specific to independent active free play. This was further complicated by the inconsistent use of terminology across studies. For instance, some studies had findings that related to issues broadly associated with independent active free play, but the authors focused the research on the more general concept of physical activity and included issues such as access to organized/supervised recreation programs [29]. These studies were removed.

Third, some studies used the term *play* in general terms (rather than the specific subtype of independent active free play). Although play outdoors was addressed in some of these studies, it often referred to sedentary (e.g., symbolic, imaginary) forms of play that did not necessarily include physical activity [30]. These studies of play in general were removed. Finally, studies of children with disabilities [31] were removed because their findings were so uniquely focused on accessibility that meaningful comparison and synthesis with other included studies was precluded.

#### Included studies

In total, 46 studies were retained for analysis. It is not possible to precisely report the total number of participants across all included studies because these details were not reported or clearly specified in all studies. Nonetheless, we were able to estimate at least 1,950 children, 1,050 parents, and 80 other adults (e.g., teachers, community members) participated in the studies (see Additional file 2: Table S1). Children in the 6–14 years age group were most frequently sampled [32-46] and parents with children aged approximately 5–11 years [17,40,43,46-53].

Of the 46 included studies, 14 were conducted in the UK [17,34-36,44,48-56], seven in the US [33,41,57-61] and seven in Australia [40,43,47,62-65]. Other studies were conducted in Canada [37,45,66,67], The Netherlands [68-70], Japan [39,71], and New Zealand [46,72]. One study was conducted in each of the following countries: Tanzania [73], Spain [74], Sweden [37], South Africa [38], France [75], India [42], and Turkey [76].

### Analysis

Adopting an interpretivist philosophical perspective, the analysis followed Paterson et al.’s [23] meta-study approach. This has four components: meta-method analysis, meta-theory analysis, and meta-data analysis, which lead to the production of a final meta-synthesis. *Meta-method analysis* was used to evaluate the methods and methodologies employed in studies (in terms of weaknesses and limitations) to identify how future research will benefit from particular research approaches. Studies were reviewed and a table created to identify research question/purpose, setting, theoretical perspective, philosophical perspective, methodology, sample characteristics, sampling procedures, data collection techniques, data analysis techniques, and validity considerations (Additional file 3: Table S2).

*Meta-theory analysis* was used to appraise the theoretical frameworks and philosophical perspectives reported in studies. Ways in which theory and philosophical perspectives may have influenced the shape and nature of the findings in the studies were evaluated.

*Meta-data analysis* is a systematic means of critically examining the studies’ findings relating to determinants of independent active free play in order to reveal their similarities and discrepancies. Two researchers reviewed the results of the included studies and extracted main findings along with exemplar quotes and organized them in a database over 22,000 words in length. A thematic analysis was conducted. Coding involved line-by-line analysis of extracted data [23,77]. Salient ‘meaning units’ were identified and themes that shared similar meanings were clustered together. A ‘long list’ of these themes was created.

*Meta-synthesis* involved the integration of interpretations from the meta-data, meta-method, and meta-theory analyses. The purpose of a meta-synthesis is to move beyond the presentation of findings and build theoretical approaches and provide direction that may extend what is currently known [23]. The meta-synthesis analysis was initially completed by two researchers and subjected to discussion and review among the other members of the research team. The steps of meta-synthesis involve a process of interpreting, theorizing, reflecting, and reviewing the previous analyses [23]. To move beyond the initial descriptive ‘long list’ of themes, an ecological framework was used to organize the results into a meaningful synthesis and a parsimonious way to represent the findings (Additional file 3: Table S2). Throughout the synthesis schematic representations of the emerging ideas and linkages were created and revised [23]. The final schematic was a theoretical framework depicting relationships between findings at different levels of social ecology.

## Results

### Meta-method analysis

Participants were generally sampled based on their residence in purposefully selected geographical areas. More specifically, in 19 studies participants were recruited based on neighborhood indicators of socioeconomic status [17,33-36,38,42,46,47,50,52,56,57,63,65,68,72,73,76]. Of these, five studies included samples from rural settings [40,51-53,55], five studies sampled individuals of a particular ethnicity [57,58,60,70,73], and three studies specifically targeted members of different generations to obtain historical perspectives [61,69,71].

Individual interviews were used in 33 studies and focus groups used in 17 studies (see Additional file 2: Table S1). Twenty-six studies used two or more data collection techniques [17,36-38,40,42-44,46,50-52,54,55,57,59-61,66-69,71,73-75]. Of these, for instance, a combination of interviews and observations were used in 10 studies [37,42,51,55,57,60,68,69,74,75]. Arts-based data collection techniques (e.g., photo elicitation, drawings, mapping) were used in conjunction with either focus group or individual interviews in eight studies [36,38,40,43,44,54,71,73]. Some form of interviewing in an outdoor setting (e.g., walkalong or on the street/intercept interviews) was used in combination with another data collection technique in six studies [50,54,59,61,66,75].

Data analysis techniques were not specified in 10 studies [36,42,43,46,49,52,53,68,74,75]. In those studies that did include some report of data analysis techniques, details provided varied considerably. Based on the information reported, it appears some form of content or thematic analysis was used in 29 studies [17,33-35,37-40,44,45,47,50,51,54-59,62,63,65-67,70-73,76]. Four studies reported the use of analysis techniques from grounded theory [41,48,60,64]. A further two studies used analytic approaches that involved the creation of narrative profiles of individuals or groups of individuals [61,68], and one used discourse analysis [32].

Details about strategies used to address validity were often lacking. The most frequently reported technique (in 13 studies) was the use of an inter-rater reliability check and/or the inclusion of more than one analyst to review the data [32,33,35,39,41,45,54,56,58,65,66,72,76]. Two studies used member-checking [54,60], which involved returning results back to participants for verification. As reported earlier, 26 studies used multiple data collection techniques, which may have allowed for methods triangulation. However, methods triangulation was only specifically claimed in three studies [60,66,73]. ‘Named’ qualitative methodologies were not specified in 27 studies (see Additional file 2: Table S1). When qualitative methodologies were specified, most popular was ethnography [42,55,61,68,75], followed by grounded theory [41,58,60], and case study [46,70]. The following methodologies were each specified once: phenomenology [73], oral history [69], action research [71], discourse analysis [32], and mixed methods [72].

### Meta-theory analysis

Four studies used a specific theory: an ecological model [47,76], Deci and Ryan’s basic psychological needs theory [45], and Hart’s rural social competence framework [55]. One study produced the dynamic theory of play choice by using grounded theory methodology [41] and another used a Foucauldian perspective [32]. While not specifying a particular theory, other studies were framed using concepts relating to the geography of outdoor spaces, including child-friendly cities [46] and various views about rural geography and the ‘myth’ of the rural idyll [17,44,50,54,58]. Others studies broadly framed the research within a range of concepts, including a classification of playground features [37], parenting styles [70], risk-taking [63], classification of residents [68], and adolescent development [64].

Philosophical perspectives/paradigms underpinning research were mentioned in four studies. Two reported a feminist or gendered perspective [68,73], one a critical perspective [32], and one worked within social constructionism [64].

## Meta-data analysis

### Child characteristics

#### Age

With increasing age children were usually more likely to be permitted by their parents or guardians to engage in independent active free play [41,45-47,49,65,74-76].

#### Competence

Children were allowed more opportunities to play if their parents perceived they were competent enough to recognize and appropriately respond to potential dangers in the outdoor environment. This was described as children being streetwise, and more streetwise children were given more freedom to play outdoors than less streetwise children [49,55,63].

#### Gender

Boys were usually permitted more freedom than girls, specifically in terms of being allowed to play outdoors more frequently, under less supervision, stay out later, and having a larger spatial range away from the family home [35,38,41,44-46,54,55,60,69,74]. In some cases, it appeared that parents’ perceptions of their children’s competence (i.e., ‘being streetwise’) could override restrictions based on gender. For example, in Valentine’s [55] study in the UK, one mother explained, “She’s [daughter] very aware of people, whereas he [son] isn’t. He’ll talk to anybody, anybody at all, he’ll speak to. I think I have to be more protective of him because he’s that way, whereas she’s different you know, she’s more of a stronger person” (p. 71).

### Parental restrictions

#### Safety concerns

The most consistent and widely reported finding was that parental concern for their children’s safety was a barrier to independent active free play. Specifically, parents were concerned about safety with respect to strangers, bullies/teenagers, and traffic [17,34,38,40,43,44,46-50,53,55,57-60,63,65,66,70,76]. For instance, reflecting concerns about strangers, one parent in O’Brien and Smith’s [48] study said, “it’s the strangers thing that worries me to death. You know, you hear so much about people whisking children away” (p. 124).

Concerns about bullies and teenagers were expressed by a participant in Pinkster and Fortuijn’s [70] study in The Netherlands, who reported: “This is not a place to raise children. The guys who hang around the neighborhood are a bad influence on kids. Kids think they are so cool, but they are involved in nasty business. They set a bad example. You don’t want your kids anywhere near those people” (p. 329). Related to traffic concerns, one mother in Jago et al.’s [56] study in the UK said, “It's not really safe to be out in the streets playing football [soccer]” (p. 474).

Retrospective and historical studies with members of different generations reported that safety concerns had increased over time, and in previous generations children had more freedom to ‘roam’ while in modern times such activity was constrained by parents [43,48,49,53,55,63,68,71]. One mother in Jenkins’ [49] study in Wales said: “When I was about fourteen or fifteen I used to spend days on the beach. I wouldn’t let my children do that at fourteen or fifteen. The dangers are the same. It’s the same sea but I just wouldn’t allow it… Yet my parents allowed us to go” (p. 383).

Conversely, some studies suggested adults’ safety concerns were not matched by children’s concerns. In Valentine’s [53] study, one girl explained, “I think I’m safe but I know, well Mum thinks that I’m not, she picks me up because of people on the streets…” (p. 82). In Thomson and Philo’s [44] research in a Scottish town, the authors concluded “children in this study were less concerned with traditional adult fears… than they were with the risk posed by other young people in their daily struggle for supremacy in their outdoor, disordered spaces” (p. 122).

Safety concerns were also raised in studies conducted in rural settings, thus questioning the ‘myth’ of the rural idyll. Although there was general consensus among participants in these studies that villages were better places to raise children than cities, these studies demonstrated stranger danger and traffic concerns were also barriers to active free play in rural areas [40,50,52-54].

#### Surveillance

Parents attempted to manage their safety concerns through some forms of surveillance, which could include setting spatial and time limits on their children (i.e., children could only go to certain locations and had to be home by a certain time), driving them to locations, only allowing them to play with friends, or insisting they carry cell phones [34,40,43,44,46-48,50,52,53,55,57,58,70]. However, some authors suggested such measures only give parents an illusion of safety because there is no risk-free approach to parenting [49]. Furthermore, in their study of a rural English village, Tucker and Matthews [54] observed youth were resistant to the idea of parental surveillance and wanted to be able to spend time outdoors away from the ‘gaze’ of adults.

### Neighborhood and physical environment

#### Fewer children in neighborhoods

A lack of children in neighborhoods meant there was an absence of friends to play with and decreased ‘safety in numbers’ [41,42,45,47,55,65,66,68,72,76]. As one participant in Jago et al.’s [56] study in the UK observed, “There are very few children who live around here. We're just in an area where there just don't seem to be that many kids” (p. 474). Another mother in the same study said, “Most of his friends don't live as close as I would like for him to be able to go wandering around the streets on his own yet” (p. 474).

#### Differences in children’s and adults’ preferences for play spaces

Eighteen studies reported differences in children’s and adults’ preferences for play spaces in neighborhoods [33-37,42-44,47,51,57,59,60,64,67,73-76]. The need for age appropriate play areas was reported, and older children and teenagers found fixed playground equipment boring [57]. Children reported preferences for flexible uses of space and locations in their neighborhoods in which they could engage in a range of different types of games and activities [51]. In contrast, parents tended to have a more constrained view of play and often focused on specific types of fixed equipment in playgrounds, such as swings, splashpads, and shade [67]. Gearin and Kahle [59] suggested that adults’ propensity for creating large-scale new spaces was at odds with children’s desires for casual open spaces.

#### Accessibility and proximity

The accessibility and proximity of play areas from the family home was reported to have a positive influence on the likelihood of children engaging in active free play [37,39,47,54,60,74,76].

#### Maintenance

The need for playground maintenance was highlighted as a factor that could positively influence children’s engagement in active free play. Children were more likely to use, or be allowed to use, playgrounds that were well maintained [50,54,59,60,65,68,74].

### Societal changes

#### Reduced sense of community

A reduced sense of community caused parents to be reluctant to allow their children to spend time outdoors [42,46,50,53,55,60,62,63,66,72,76]. A participant in Holt et al.’s [66] study in Canada said, “…people don’t have the time to meet people or be a community… And I think that’s sad to see, most people don’t know their neighbors now… you don’t know the names of people two or three houses down” (p. 9).

Accordingly, reviving independent active free play required the creation of an increased sense of community that involved people knowing their neighbors, spending more time outdoors in their neighborhoods, and organizing community-oriented events [53,57,63,66,72]. As Little [63] suggested, increasing children’s independent active free play “requires change at a community and societal level in providing safe spaces for play where children can exercise their agency and learn to manage risks for themselves” (p. 14).

#### Good parenting ideal

The concept of the ‘good parenting’ ideal was coded as a societal level factor that has influenced children’s independent active free play. The good parenting ideal refers to parents’ perceptions of what other parents in their society would perceive to be good parenting. For instance, some parents felt the need to monitor their children at all times in order to be seen as good parents, whereas they perceived allowing children to roam free was a feature of poor parenting [17,39,46,55,58,63,70]. Parents of lower socio economic status were more likely to allow their children to roam free than parents of higher socio economic status [17,46,70], further suggesting the good parenting ideal was related to societal level factors. A different concept of a good parenting ideal was reported in a study from Japan [39], whereby parents’ emphasis on their children’s academic performance meant children had to spend free-time studying, thus reducing their opportunities to engage in independent active free play.

#### Changing roles of parents

The changing roles of parents and, in particular, the increased number of mothers in the workforce, were noted as factors which reduced children’s engagement in independent active free play in four studies [62,68,71,72]. One participant in Witten et al.’s [72] study in New Zealand said, “Maybe more people are working these days, cause, you know, things are more expensive so where back then mothers would, you know, most of them stay home…” (p. 6). Again, this societal level factor related to factors at the parent level.

#### Privatization of play spaces and playtime

Societal and economic forces meant that children’s play spaces and playtime have become increasingly privatized [17,43,52,61,65,72]. For instance, public areas in which children could play outdoors have become the property of private landowners and/or contested spaces in which children’s needs had become subservient to the needs of adults [44,50,52-54,57,61]. Valentine and McKendrick [17] argued a consequence of the privatization of playtime is that children “are spending more time under adult supervision, either while playing in the garden, or at institutionally-based play activities” (p. 231).

### Policy issues

#### Give children voice

No studies explicitly examined the influence of policies governing children’s active free play. However, several studies broadly considered issues relating to policy, and the most consistent point, usually presented as a conclusion or future direction, was the need to include children in planning decisions to help ensure their ‘voice’ would be heard [33,38,40,44,46,50,54,55,59,74]. For instance, Burke [36] urged planners to “see with the eyes of children and to allow for play opportunities that do not necessarily immediately serve adult interests and allay adult concerns” (p. 50).

### Meta-synthesis

**Figure 2:**
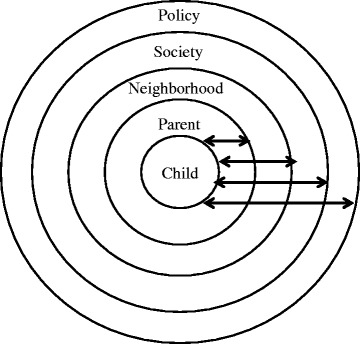
**Meta-synthesis of factors influencing children’s active free play outdoors.**

Taken together, the studies reviewed indicated a range of factors at different levels of social ecology influence independent active free play. Broadly using concepts from Bronfenbrenner’s ecological framework [78,79] to depict ways in which the findings fitted together, this synthesis demonstrates that, in addition to the organization of findings from more proximal/micro to more distal/macro levels, there were a series of reciprocal relationships between factors at different levels of social ecology. These relationships are depicted by the bi-directional arrows on Figure 2.

Children are located at the centre of the model, and their personal characteristics (age, competence, gender) moderate their opportunities to engage in independent active free play. Parents have the most direct influence on their children’s play (reflected by the fact that parents are the most proximal ecological influence on the child). Primarily due to their concern for their children’s safety, parents set limits on the extent to which they can engage in independent active free play. However, older children, boys, and more competent (streetwise) children are generally given more freedom.

The parent level is also influenced by neighborhood level factors associated with children’s engagement in independent active free play. For instance, the absence of other children in a neighborhood means there are limited opportunities for children to have safety in numbers. Presumably, when there are fewer children in a neighborhood parents are less willing to let their children play unsupervised.

Parent and neighborhood level factors have, in turn, been influenced by broader societal changes. A reduced sense of community restricts the extent to which parents will let their children play outdoors. Parents’ supervisory behaviors are further influenced by societal expectations for good parenting. Some parents perceive that they are judged by the extent to which they are seen to be in control of their children and not allowing them to roam free without adult supervision. Simultaneously, changing employment patterns have restricted opportunities for parents to spend time with their children. Perhaps as a consequence of some parents working long hours and their perceived need to have their children supervised, they are more likely to put their children in private and supervised/organized forms of physical activity. This seems particularly relevant to parents who have sufficient income to pay for such organized programs.

Hence, parents are influenced by factors at different levels of social ecology. Simultaneously, parents’ actions also influence these factors in a reciprocal manner. If parents restrict their children’s freedom to spend time outdoors there are fewer children to play with, thus further restricting parents’ willingness to let their children go out and play due to the absence of safety in numbers. If parents are more likely to drive their children than let them walk due to concerns about road traffic, they therefore contribute to increasing the amount of road traffic. Combined, these ‘social traps’ [18,46] reduce the number of people who spend time outdoors in a neighborhood, presumably reducing a sense of community and social cohesion and increasing perceived safety concerns. These examples, and the model presented in Figure 2, highlight the reciprocal nature of factors at multiple levels of social ecology that influence children’s independent active free play.

## Discussion

This is the first meta-study produced by completing a systematic review of qualitative research examining determinants of independent active free play research in children. It includes a larger number of studies than previous reviews [18-20] and draws together findings from a fragmented body of literature spanning several academic disciplines. The findings contribute to a knowledge base for guiding further studies of children’s independent active free play and have implications for the use of methods and theory for qualitative studies in the future.

Consistent with the findings of Carver et al.’s [19] narrative review, the most widely reported finding in the literature was parents’ safety concerns restrict children’s independent active free play [17,34,38,40,43,44,46-50,53,55,57-60,63,65,66,70,76]. Though children may not share the same safety concerns as their parents [44,53], parents’ safety concerns emerged as the primary barrier to children’s active free play.

In a recent systematic review, Ding, Sallis, Kerr, Lee, and Rosenberg [80] showed associations between traffic safety and physical activity were more consistent than those with crime-related safety. They further observed quantitative measures of safety were often crude and rarely validated, and more sophisticated measures are needed. The findings of the current study provide a more sophisticated understanding of safety concerns. For instance, the fact that older, more competent (streetwise) children are given the most freedom to play is a reflection of parents’ appraisals of their children’s ability to handle safety concerns. Thus, child-level characteristics may, to some extent, ameliorate parents’ concerns about the safety of their neighborhoods. Parents’ safety concerns were, in turn, influenced by a reduced sense of community and their societal expectations of what good parenting entails. We suggest, therefore, that merely assessing safety (whether objectively or subjectively) may not be sufficient for understanding parents’ restrictions on their children’s play. It is important to also consider the other factors that influence, and are influenced by, parents’ safety concerns. Specifically, including assessments of parents’ perceptions of their children’s competence along with their supervisory/parenting style would provide more sophisticated ways to measure associations between safety and active free play.

It is important to note the majority of studies included in this review were conducted in developed countries, where crimes rates are generally in decline. In the UK, in 1995, 5.3% of adults aged 16 and over were a victim of violent crime compared with 2.6% in 2012/2013 [81]. In the US, the 2012 estimated violent crime total was 12.2% below the 2003 level [82]. In Canada, reported crime in 2009 was at its lowest point since 1973, and 93% of respondents to the General Social Survey reported they were satisfied with their personal safety from crime [83]. An exception is Australia, where while the number of recorded sexual assaults and robberies decreased, reported kidnapping and abductions increased from 478 cases in 1996 to 670 cases in 2011 [84].

The issue may be that public perception of crime is higher than any specific threat to children. For instance, according to a Royal Canadian Mounted Police report, cases of children’s so-called ‘abduction by strangers’ (a category which actually includes relatives and close friends) ranged from 42 cases in 1998 to 35 cases in 2002 [85]. The authors observed the common assumption that an offender ‘comes out of nowhere and snatches a child’ is rare, and most often the offender is a family member or an acquaintance of the child and/or family. Hence, parents’ safety concerns are not consistent with the objective evidence. Rather, public anxiety about stranger abductions is intensified by sensationalist media coverage [85].

The current review provides some suggestions for future interventions and policies that may increase active free play. Traffic calming, crime prevention, and community policing strategies offer one set of alternatives for addressing safety concerns. From a population health perspective, our findings suggest that community-level interventions targeted at building social cohesion are required [53,63,66,72]. There were also calls for the inclusion of children in community planning decisions to accommodate their needs [33,38,40,44,46,50,54,55,59,74]. Avenues to engage children in planning decisions are required for creating community-oriented change that caters to children’s needs. This is important because children and adults appear to have different preferences for the provision of play areas.

Furthermore, it was notable that no studies included in this review explicitly examined the influence of planning and policy decisions on children’s active free play, even though many municipalities have policies in place that specifically discourage children’s use of outdoor space [86,87]. The findings of the current review clearly point to the need for community-based intervention and policy initiatives, ideally including consultative processes with children, to address the barriers presented by parental safety concerns.

From a methods perspective, strengths of the published literature included careful sampling of participants in geographical locations considering a range of social and demographic factors, and the use of a range of data collection techniques (often in conjunction). Main weaknesses in the studies were that ‘named’ methodologies were rarely specified, data analysis procedures were often poorly/not explained, and few studies included attempts to address validity. These weaknesses should be addressed in future work in order to produce more rigorous research designs. One useful strategy for designing qualitative studies is the ‘armchair walkthrough’ [88], which refers to a process of thinking through the methodological trajectory of a research project. This approach helps authors establish the methodological congruence of their studies by considering philosophical, theoretical, and methodological features of research design.

In terms of theories, the main conclusions drawn are that a focus on understanding children’s perspectives and children’s ‘geographies’ has been prevalent in the literature, and a handful of studies have used ecological perspectives. However, the majority of studies were descriptive with theoretical approaches rarely being reported and therefore it was difficult to establish how theoretical perspectives have shaped knowledge in this area. Though qualitative studies are often descriptive, it is possible to use theory within such designs to advance the generation of knowledge. For instance, Sandelowski [89] explained that theory can be used to establish the conceptual context for a qualitative study, to guide the data analysis, or to interpret findings in a discussion. The qualitative literature examining children’s independent active free play could be improved by incorporating theory into research designs to provide a heuristic for studying concepts which may meaningfully explain and predict behaviors, experiences, and outcomes and provide a guide for action/intervention. Given the way we were able to organize and synthesize the findings of this review, it would appear that ecological theories may be useful in the future. Furthermore, contextualized theories of parenting styles and strategies may be useful for examining relationships between parent-, child-, and social-level factors [90].

The results of this study help identify several important directions for future research. For instance, sophisticated analyses of the different types of safety concerns children and parents perceive, how they may differ between children and parents, and how they are related to independent active free play and other forms of physical activity are required. The existing literature could also be expanded by studies of children’s perceptions of planning decisions (e.g., around new playgrounds) and, when possible, studies of instances when children have actually been consulted in planning decisions. Community-based participatory forms of research could be used to investigate such issues. Historical research, including multiple generations of families, may reveal more information about changes in children’s play over time. There also remains a need for more analysis of play in rural settings. Studies that examine issues of multiple levels of social ecology, and relationships therein, would make important contributions to the literature. Finally, the majority of studies to date have been conducted in western developed countries, and there is a need to understand more about play among children in developing countries.

At the procedural level, the quality of this meta-study can be judged based on its adherence to principles of design. We completed an exhaustive search of the literature across disciplines and considered more studies than have been included in previous reviews [18-20]. It is difficult to ensure every relevant study is found in any type of systematic review [91]. With a literature as diverse as that reviewed here, produced by authors operating and publishing in a range of disciplines, there is a risk that some studies may have been inadvertently excluded. This is a potential limitation of all systematic reviews [23], but the inclusion of an information systems expert in the search and retrieval of studies helps mitigate this concern [92]. Another challenge we encountered was the vast variation in terminology employed across studies/disciplines in the absence of clear definitions (in particular, studies that were framed from a general physical activity or general play perspective in the absence of clear definition of the terms and delineated focus on active free play). To create a more coherent body of literature in this area in the future, authors must carefully consider the terminology and provide specific definitions of the construct they are investigating.

Another possible limitation to consider relates to our focus on independent active free play that occurs outside of supervised/adult-directed settings. There is qualitative research examining play during (for example) recess, after-school programs, and in kindergarten settings (e.g., [27,28]). Play in these contexts provides important developmental opportunities and contributes to children’s overall engagement in physical activity. Given the large number of studies we included in the current review, it was outside our scope to include the studies of play in supervised/adult-directed settings, and the element of adult involvement would likely have produced quite different findings to the issues revealed in our current review. Therefore, this remains an area that should be subjected to systematic review in the future in order to provide more information about supervised/adult-directed play. Indeed, given that parents’ safety concerns limit their children’s involvement in independent active free play, it is likely that supervised/adult-directed settings will become increasingly important in the future.

## Conclusions

This comprehensive meta-study helps establish a knowledge base of qualitative research examining children’s independent active free play by synthesizing a previously fragmented set of studies. The findings show parents’ perceived safety concerns are the primary barrier to children’s independent active free play. These safety concerns are moderated by child-level factors (age, competence, gender) and broader societal level issues. Interventions should focus on community-level solutions that include children’s perspectives. The reviewed studies used a range of data collection techniques, but other methodological details were often inadequately reported. The theoretical sophistication of research in this area could be improved. To this end, the synthesis reported in this study provides a framework for guiding future research.

## Endnote

^a^We wish to emphasize that we removed studies in adult-directed settings (e.g., school playgrounds, supervised after-school programs). We realize, however, that some children’s play in informal settings (e.g., greenspaces) may include some adult supervision, but these informal settings lack the often strict rules that are in place in adult-directed settings (e.g., rules imposed by teachers about access to play areas).

## Additional files

Additional file 1:
**Search strategies.**
Additional file 2: Table S1. Meta-Method Extraction. Additional file 3: Table S2. Factors That Influence Children’s Engagement in Active Free Play.
