# Supplementary material for: A meta-study of qualitative research examining determinants of children’s independent active free play
Source: Int J Behav Nutr Phys Act. 2015 Jan 24;12:5. doi: 10.1186/s12966-015-0165-9 (PMC4318368; doi:10.1186/s12966-015-0165-9)
Supplement: Additional file 1: — Search strategies. [file 12966_2015_165_MOESM1_ESM.docx]

**Additional file 1**: **Search strategies**

| **EBSCO Sport Discus 1975-Present [Same strategy for Academic Search Complete and Child Development & Adolescent Studies]** | **Ovid MEDLINE(R) In-Process & Other Non-Indexed Citations, Ovid MEDLINE(R) Daily and Ovid MEDLINE(R) 1946 to Present** | **Ovid ERIC – 1965-Present** |
| --- | --- | --- |
| S7 S1 and S2 and S6  S6 S3 or S4 or S5  S5 SU play  S4 ( skipping or "skip* rope" or "jump rope*" or bike* or biking or cycling or bicyling or tricycle* or tricycling ) AND play*  S3 (active or informal or free or casual or "out* of school" or unstructured or spontaneous or outdoor or "pick up" or wilderness or nature or recess or lunchtime* or "lunch time*" or recreational) n10 (play* or game* or "physical activity" ) or exergame* or esport* or hopscotch or tag or playground*  S2 TI (child* or adolescen* or teen* or youth or p#diatric*) or SU (child* or adolescen* or teen* or youth or p#diatric*)  S1 qualitative OR ethnol* OR ethnog* OR ethnonurs* OR emic OR etic OR leininger OR noblit OR "field note*" OR "field record*" OR fieldnote* OR "field stud*" or "participant observ*" OR "participant observation*" OR hermaneutic* OR phenomenolog* OR "lived experience*" OR heidegger* OR husserl* OR "merleau-pont*" OR colaizzi OR giorgi OR ricoeur OR spiegelberg OR "van kaam" OR "van manen" OR "grounded theory" OR "constant compar*" OR "theoretical sampl*" OR glaser AND strauss OR "content analy*" OR "thematic analy*" OR narrative* OR "unstructured categor*" OR "structured categor*" OR "unstructured interview*" OR "semi-structured interview*" OR "maximum variation*" OR snowball OR audio* OR tape* OR video* OR metasynthes* OR "meta-synthes*" OR metasummar* OR "meta-summar*" OR metastud* OR "meta-stud*" OR "meta-ethnograph*" OR metaethnog* OR "meta-narrative*" OR metanarrat* OR " meta-interpretation*" OR metainterpret* OR "qualitative meta-analy*" OR "qualitative metaanaly*" OR "qualitative metanaly*" OR "purposive sampl*" OR "action research" OR "focus group*" or "photo voice" or photovoice | 1. exp "Play and Playthings"/  2. (((active or informal or free or casual or "out* of school" or unstructured or spontaneous or outdoor or "pick up" or wilderness or nature or recess or lunchtime* or lunch time* or recreational) adj10 (play* or game* or "physical activity")) or exergame* or esport* or hopscotch or tag).mp. [mp=title, abstract, subject headings, heading word, drug trade name, original title, device manufacturer, drug manufacturer, device trade name, keyword]  3. ((skipping or "skip* rope" or "jump rope*" or bike* or biking or cycling or bicyling or tricycle* or tricycling) and play*).mp. [mp=title, abstract, subject headings, heading word, drug trade name, original title, device manufacturer, drug manufacturer, device trade name, keyword]  4. playground*.mp.  5. 1 or 2 or 3 or 4  6. (child* or adolescen* or teen* or youth or p?ediatric*).mp,jw,nw.  7. 5 and 6  8. exp qualitative research/  9. exp Nursing Methodology Research/  10. anthropology, cultural/  11. exp focus groups/  12. qualitative.mp.  13. (ethnol$ or ethnog$ or ethnonurs$ or emic or etic).mp.  14. (leininger$ or noblit).mp. or hare.ti,ab.  15. (field note$ or field record$ or fieldnote$ or field stud$).mp.  16. (participant$ adj3 observ$).mp.  17. (nonparticipant$ adj3 observ$).mp.  18. (non participant$ adj3 observ$).mp.  19. (hermeneutic$ or phenomenolog$ or lived experience$).mp.  20. (heidegger$ or husserl$ or merleau-pont$).mp.  21. (colaizzi$ or giorgi$).mp.  22. (ricoeur or spiegelberg$).mp.  23. (van kaam$ or van manen).mp.  24. (Grounded adj5 theor$).mp.  25. (constant compar$ or theoretical sampl$ or triangulat$).ti,ab.  26. (glaser and strauss).mp.  27. (content analys$ or thematic analys$ or narrative analys$).mp.  28. (unstructured categor$ or structured categor$).mp.  29. (unstructured interview$ or semi-structured interview$ or semistructured interview$).mp.  30. (maximum variation or snowball).mp.  31. (audio$ or video$ or tape$).mp. adj5 (interview$.mp. or px.fs.)  32. (metasynthes$ or meta-synthes$ or metasummar$ or meta-summar$ or metastud$ or meta-stud$).ti,ab.  33. (meta-ethnog$ or metaethnog$ or meta-narrat$ or metanarrat$ or meta-interpret$ or metainterpret$).mp.  34. (qualitative adj5 meta-analy$).mp.  35. (qualitative adj5 metaanaly$).mp.  36. purposive sampl$.mp.  37. action research.mp.  38. focus group$.mp.  39. (exp interview/ or exp interview as topic/) and px.fs.  40. (theme*.ti,ab. or experience*.ti. or audio*.ti,ab. or video*.ti,ab. or recording.ti,ab. or exp Tape Recording/ or exp Video Recording/) and px.fs.  41. (experience*.ti. or theme*.ti,ab.) and ((audio* or video* or recording).ti,ab. or exp Tape Recording/ or exp Video Recording/)  42. interview*.ti,ab. and (views or perception* or belief* or attitude* or barrier* or facilitat* or perspective*).mp.  43. or/8-42  44. 7 and 43 | 1. play/ or playground activities/  2. (((active or informal or free or casual or "out* of school" or unstructured or spontaneous or outdoor or "pick up" or wilderness or nature or recess or lunchtime* or lunch time* or recreational) adj10 (play* or game* or "physical activity")) or exergame* or esport* or hopscotch or tag).mp. [mp=abstract, title, heading word, identifiers]  3. ((skipping or "skip* rope" or "jump rope*" or bike* or biking or cycling or bicyling or tricycle* or tricycling) and play*).mp. [mp=abstract, title, heading word, identifiers]  4. playground*.mp.  5. 1 or 2 or 3 or 4  6. (child* or adolescen* or teen* or youth or p?ediatric*).mp,jx.  7. 5 and 6  8. exp Qualitative Research/  9. exp phenomenology/ or exp hermeneutics/  10. exp Action Research/  11. exp ETHNOGRAPHY/  12. exp Focus Groups/  13. exp Content Analysis/  14. exp Field Studies/  15. qualitative.mp.  16. (ethno$ or emic or etic).mp.  17. (leininger$ or noblit or hare).ti,ab.  18. (field note$ or field record$ or fieldnote$ or field stud$).mp.  19. (participant$ adj3 observ$).mp.  20. (nonparticipant$ adj3 observ$).mp.  21. (non participant$ adj3 observ$).mp.  22. (hermeneutic$ or phenomenolog$ or lived experience$).mp.  23. (heidegger$ or husserl$ or merleau-pont$).mp.  24. (colaizzi$ or giorgi$).mp.  25. (ricoeur or spiegelberg$).mp.  26. (van kaam$ or van manen).mp.  27. (Grounded adj5 theor$).mp.  28. (constant compar$ or theoretical sampl$ or triangulat$).ti,ab.  29. (glaser or strauss or chenitz).mp.  30. (content analys$ or thematic analys$ or narrative analys$).mp.  31. (unstructured categor$ or structured categor$).mp.  32. (unstructured interview$ or semi-structured interview$ or semistructured interview$).mp.  33. (maximum variation or snowball).mp.  34. (audiorecord$ or taperecord$ or videorecord$ or videotap$).mp.  35. ((audio or tape or video$) adj5 record$).mp.  36. ((audio$ or video$ or tape$) adj5 interview$).mp.  37. (metasynthes$ or meta-synthes$ or metasummar$ or meta-summar$ or metastud$ or meta-stud$).ti,ab.  38. (meta-ethnog$ or metaethnog$ or meta-narrat$ or metanarrat$ or meta-interpret$ or metainterpret$).mp.  39. (qualitative adj5 meta-analy$).mp.  40. (qualitative adj5 metaanaly$).mp.  41. purposive sampl$.mp.  42. action research.mp.  43. focus group$.mp.  44. or/8-43  45. 7 and 44 |
| **Ovid PsycInfo 1806-Present** | **Ovid EMBASE, 1974-Present** | **ProQuest Sociological Abstracts [Same strategy for ProQuest Dissertations & Theses Full-text and Physical Education Index]** |
| 1. (((active or informal or free or casual or "out* of school" or unstructured or spontaneous or outdoor or "pick up" or wilderness or nature or recess or lunchtime* or lunch time*) adj10 (play* or game* or "physical activity")) or exergame* or esport* or hopscotch or tag).mp. [mp=title, abstract, heading word, table of contents, key concepts, original title, tests & measures]  2. ((skipping or "skip* rope" or "jump rope*" or bike* or biking or cycling or bicyling or tricycle* or tricycling) and play*).mp. [mp=title, abstract, heading word, table of contents, key concepts, original title, tests & measures]  3. playground*.mp.  4. 1 or 2 or 3  5. (child* or adolescen* or teen* or youth or p?ediatric*).mp,jx.  6. 4 and 5  7. qualitative study.md.  8. exp QUALITATIVE RESEARCH/  9. phenomenology/ or constructivism/ or hermeneutics/  10. exp ETHNOGRAPHY/  11. exp Content Analysis/  12. qualitative.mp.  13. (ethno$ or emic or etic).mp.  14. (leininger$ or noblit or hare).ti,ab.  15. leininger m$.cu.  16. noblit g$.cu.  17. hare r$.cu.  18. (field note$ or field record$ or fieldnote$ or field stud$).mp.  19. (participant$ adj3 observ$).mp.  20. (nonparticipant$ adj3 observ$).mp.  21. (non participant$ adj3 observ$).mp.  22. (hermeneutic$ or phenomenolog$ or lived experience$).mp.  23. (heidegger$ or husserl$ or merleau-pont$).mp,cu.  24. (colaizzi$ or giorgi$).mp,cu.  25. (ricoeur or spiegelberg$).mp,cu.  26. (van kaam$ or van manen).mp,cu.  27. (Grounded adj5 theor$).mp.  28. (constant compar$ or theoretical sampl$ or triangulat$).ti,ab.  29. (glaser or strauss).mp.  30. glaser b$.cu.  31. strauss a$.cu.  32. (content analys$ or thematic analys$ or narrative analys$).mp.  33. (unstructured categor$ or structured categor$).mp.  34. (unstructured interview$ or semi-structured interview$ or semistructured interview$).mp.  35. (maximum variation or snowball).mp.  36. (audiorecord$ or taperecord$ or videorecord$ or videotap$).mp.  37. ((audio or tape or video$) adj5 record$).mp.  38. ((audio$ or video$ or tape$) adj5 interview$).mp.  39. (metasynthes$ or meta-synthes$ or metasummar$ or meta-summar$ or metastud$ or meta-stud$).ti,ab.  40. (meta-ethnog$ or metaethnog$ or meta-narrat$ or metanarrat$ or meta-interpret$ or metainterpret$).mp.  41. (qualitative adj5 meta-analy$).mp.  42. (qualitative adj5 metaanaly$).mp.  43. purposive sampl$.mp.  44. action research.mp.  45. focus group$.mp.  46. or/7-45  47. 6 and 46 | 1. exp Play/  2. (((active or informal or free or casual or "out* of school" or unstructured or spontaneous or outdoor or "pick up" or wilderness or nature or recess or lunchtime* or lunch time* or recreational) adj10 (play* or game* or "physical activity")) or exergame* or esport* or hopscotch or tag).mp. [mp=title, abstract, subject headings, heading word, drug trade name, original title, device manufacturer, drug manufacturer, device trade name, keyword]  3. ((skipping or "skip* rope" or "jump rope*" or bike* or biking or cycling or bicyling or tricycle* or tricycling) and play*).mp. [mp=title, abstract, subject headings, heading word, drug trade name, original title, device manufacturer, drug manufacturer, device trade name, keyword]  4. playground*.mp.  5. 1 or 2 or 3 or 4  6. (child* or adolescen* or teen* or youth or p?ediatric*).mp,jw,nw.  7. 5 and 6  8. qualitative.mp.  9. (ethnol$ or ethnog$ or ethnonurs$ or emic or etic).mp.  10. (leininger$ or noblit or hare).ti,ab.  11. (field note$ or field record$ or fieldnote$ or field stud$).mp.  12. (participant$ adj3 observ$).mp.  13. (nonparticipant$ adj3 observ$).mp.  14. (non participant$ adj3 observ$).mp.  15. (hermeneutic$ or phenomenolog$ or lived experience$).mp.  16. (heidegger$ or husserl$ or merleau-pont$).mp.  17. (colaizzi$ or giorgi$).mp.  18. (ricoeur or spiegelberg$).mp.  19. (van kaam$ or van manen).mp.  20. (Grounded adj5 theor$).mp.  21. (constant compar$ or theoretical sampl$ or triangulat$).ti,ab.  22. (glaser and strauss).mp.  23. (content analys$ or thematic analys$ or narrative analys$).mp.  24. (unstructured categor$ or structured categor$).mp.  25. (unstructured interview$ or semi-structured interview$ or semistructured interview$).mp.  26. (maximum variation or snowball).mp.  27. ((audio$ or video$ or tape$) adj5 interview$).mp.  28. (metasynthes$ or meta-synthes$ or metasummar$ or meta-summar$ or metastud$ or meta-stud$).ti,ab.  29. (meta-ethnog$ or metaethnog$ or meta-narrat$ or metanarrat$ or meta-interpret$ or metainterpret$).mp.  30. (qualitative adj5 meta-analy$).mp.  31. (qualitative adj5 metaanaly$).mp.  32. purposive sampl$.mp.  33. action research.mp.  34. focus group$.mp.  35. (photovoice or photo voice).mp. [mp=title, abstract, subject headings, heading word, drug trade name, original title, device manufacturer, drug manufacturer, device trade name, keyword]  36. or/8-35  37. 7 and 36 | ((ti(play) OR all(((active OR informal OR free OR casual OR "out* of school" OR unstructured OR spontaneous OR outdoor OR "pick up" OR wilderness OR nature OR recess OR lunchtime* OR "lunch time*" OR recreational) n10 (play* OR game* OR "physical activity")) OR exergame* OR esport* OR hopscotch OR tag OR playground* OR "skip* rope" OR "jump rope*" OR tricycle* OR tricycling OR rollerblading OR rollerskating OR skating OR skiing OR snowboarding OR scooter* OR softball OR baseball OR swim* OR "hide and go seek" OR swings OR slides OR climb* OR sledge* OR sledding OR "snowball fight*" OR "rough and tumble" OR "splash pads" OR "spray pads" OR "wading pool*" OR toboggan* OR bike* OR biking OR bicyling)) AND ALL(child* OR adolescen* OR teen* OR youth OR pediatric* OR paediatric*)) AND (all(qualitative OR ethnol* OR ethnog* OR ethnonurs* OR emic OR etic OR leininger OR noblit OR "field note*" OR "field record*" OR fieldnote* OR "field stud*" OR "participant observ*" OR "participant observation*" OR hermaneutic* OR phenomenolog* OR "lived experience*" OR heidegger* OR husserl*) OR all("merleau-pont*" OR colaizzi OR giorgi OR ricoeur OR spiegelberg OR "van kaam" OR "van manen" OR "grounded theory" OR "constant compar*" OR "theoretical sampl*" OR glaser AND strauss OR "content analy*" OR "thematic analy*" OR narrative* OR "unstructured categor*" OR "structured categor*") OR all("unstructured interview*" OR "semi-structured interview*" OR "maximum variation*" OR snowball OR audio* OR tape* OR video* OR metasynthes* OR "meta-synthes*" OR metasummar* OR "meta-summar*" OR metastud* OR "meta-stud*" OR "meta-ethnograph*" OR metaethnog* OR "meta-narrative*" OR metanarrat* OR " meta-interpretation*" OR metainterpret* OR "qualitative meta-analy*" OR "qualitative metaanaly*" OR "qualitative metanaly*" OR "purposive sampl*" OR "action research" OR "focus group*" OR "photo voice" OR photovoice)) |
| Elsevier Scopus, 1960-Current | ISI Web of Science Core Collection, 1900-Current |  |
| (TITLE-ABS-KEY((active OR informal OR free OR casual OR "out* of school" OR unstructured OR spontaneous OR outdoor OR "pick up" OR wilderness OR nature OR recess OR lunchtime* OR "lunch time*" OR recreational) AND (play* OR game*)) OR TITLE-ABS-KEY(exergame* OR esport* OR hopscotch OR tag OR playground* OR skipping OR "skip* rope" OR "jump rope*" OR bike* OR biking OR cycling OR bicyling OR tricycle* OR tricycling OR rollerblading OR rollerskating OR skating OR skiing OR snowboarding OR scooter* OR softball OR baseball OR swim* OR "hide and go seek" OR swings OR slides OR climb* OR sledge* OR sledding OR "snowball fight*" OR "rough and tumble" OR "splash pads" OR "spray pads" OR "wading pool*" OR toboggan*) OR TITLE("physical* activ*" OR su "physical* activ*" OR play) OR KEY(play)) AND (TITLE(child* OR adolescen* OR teen* OR youth OR pediatric* OR paediatric*) OR KEY(child* OR adolescen* OR teen* OR youth OR pediatric* OR paediatric*)) AND ((qualitative OR ethnol* OR ethnog* OR ethnonurs* OR emic OR etic OR leininger OR noblit OR "field note*" OR "field record*" OR fieldnote* OR "field stud*" OR "participant observ*" OR "participant observation*" OR hermaneutic* OR phenomenolog* OR "lived experience*" OR heidegger* OR husserl* OR "merleau-pont*" OR colaizzi OR giorgi OR ricoeur OR spiegelberg OR "van kaam" OR "van manen" OR "grounded theory" OR "constant compar*" OR "theoretical sampl*" OR glaser AND strauss OR "content analy*" OR "thematic analy*" OR narrative* OR "unstructured categor*" OR "structured categor*" OR "unstructured interview*" OR "semi-structured interview*" OR "maximum variation*" OR snowball OR audio* OR tape* OR video* OR metasynthes* OR "meta-synthes*" OR metasummar* OR "meta-summar*" OR metastud* OR "meta-stud*" OR "meta-ethnograph*" OR metaethnog* OR "meta-narrative*" OR metanarrat* OR " meta-interpretation*" OR metainterpret* OR "qualitative meta-analy*" OR "qualitative metaanaly*" OR "qualitative metanaly*" OR "purposive sampl*" OR "action research" OR "focus group*" OR "photo voice" OR photovoice)) | #4 #1 AND #2 AND #3 AND #4   \| #3 \| TI=(child* or adolescen* or teen* or youth or pediatric* or paediatric*) \| \| --- \| --- \|   #2 TS=(qualitative OR ethnol* OR ethnog* OR ethnonurs* OR emic OR etic OR leininger OR noblit OR "field note*" OR "field record*" OR fieldnote* OR "field stud*" or "participant observ*" OR "participant observation*" OR hermaneutic* OR phenomenolog* OR "lived experience*" OR heidegger* OR husserl* OR "merleau-pont*" OR colaizzi OR giorgi OR ricoeur OR spiegelberg OR "van kaam" OR "van manen" OR "grounded theory" OR "constant compar*" OR "theoretical sampl*" OR glaser AND strauss OR "content analy*" OR "thematic analy*" OR narrative* OR "unstructured categor*" OR "structured categor*" OR "unstructured interview*" OR "semi-structured interview*" OR "maximum variation*" OR snowball OR audio* OR tape* OR video* OR metasynthes* OR "meta-synthes*" OR metasummar* OR "meta-summar*" OR metastud* OR "meta-stud*" OR "meta-ethnograph*" OR metaethnog* OR "meta-narrative*" OR metanarrat* OR " meta-interpretation*" OR metainterpret* OR "qualitative meta-analy*" OR "qualitative metaanaly*" OR "qualitative metanaly*" OR "purposive sampl*" OR "action research" OR "focus group*" or "photo voice" or photovoice)  #1 TS=( (active or informal or free or casual or "out* of school" or unstructured or spontaneous or outdoor or "pick up" or wilderness or nature or recess or lunchtime* or "lunch time*" or recreational) same (play* or game*) or exergame* or esport* or hopscotch or tag or playground* or skipping or "skip* rope" or "jump rope*" or bike* or biking or cycling or bicyling or tricycle* or tricycling or rollerblading or rollerskating or skating or skiing or snowboarding or scooter* or softball or baseball or swim* or "hide and go seek" or swings or slides or climb* or sledge* or sledding or "snowball fight*" or "rough and tumble" or "splash pads" or "spray pads" or "wading pool*" or toboggan*) or TI="physical* activ*" |  |
