# Supplementary material for: A meta-study of qualitative research examining determinants of children’s independent active free play
Source: Int J Behav Nutr Phys Act. 2015 Jan 24;12:5. doi: 10.1186/s12966-015-0165-9 (PMC4318368; doi:10.1186/s12966-015-0165-9)
Supplement: Additional file 2: Table S1. — Meta-Method Extraction. [file 12966_2015_165_MOESM2_ESM.docx]

Additional file 2 Table S1 Meta-Method Extraction

| **Study** | **Research question(s)/ Purpose** | **Setting** | **Theoretical Perspective** | **Philosophical Perspective** | **Methodology** | **Sample Characteristics** | **Sampling Strategy** | **Data Collection Techniques** | **Data Analysis Techniques** | **Validity Considerations** |
| --- | --- | --- | --- | --- | --- | --- | --- | --- | --- | --- |
| Andrews et al. (2014) | Parents’ perceptions of raising their children in outer suburbs. | Melbourne, Australia. | Not specified. | Not specified. | Not specified. | 22 parents aged 26-42 years (1 male, 21 female). | Purposeful sampling to recruit parents with at least on pre-school aged child who had lived in new suburbs for at least 12 months. | Individual interviews. | Thematic analysis. | Not specified. |
| Alexander et al. (2014) | To examine knowledge formations, vales, and normative assumptions that underlie public health discourse about children’s play. | Canada. | Foucault. | Foucault/critical. | Critical discourse analysis. | Not applicable (documentary analysis). | Not applicable. | 150 documents from six prominent Canadian health-related organizations were obtained and reviewed. | Thematic analysis. Discourse analysis | Third party inter-rater reliability check. |
| Berinstein & Magalhaes (2009) | To gain an understanding of the essence of play to experience children. | Zanzibar, Tanzania. | Not specified. | Feminist perspective mentioned. | Phenomenological approach using photovoice. | 16 students (4 female) aged 10-13 years from varying socioeconomic backgrounds, predominantly of the Muslim religion. | Selected by school headmistress. Criteria not reported. | Photovoice process over 4 weeks followed by focus group. | Thematic analysis. | Methodological triangulation. |
| Blakely (1994) | Explores parents' conceptions of dangers in their changing neighborhoods in relationship to the activities of the nine- to eleven-year-old children. | New York City, US. | Not specified. | Not specified. | “Based on tradition of grounded theory” (p. 16). | 32 mothers and 10 fathers. (16 Hispanic mothers, 16 non-Hispanic mothers, 5 Hispanic fathers, 5 non-Hispanic fathers). | Purposeful sampling from two multi-ethnic areas. | Two-part interview with open- and closed-ended questions. | Content analysis to identify themes, categories, and concepts. | Inter-coder reliability by two independent judges. |
| Brockman, Fox, & Jago (2011) | What are 10-11 year old children's perceptions of play? How much of this is 'active play'? What are the contexts of children's active play? What do they do in their active play? Where do they engage in active play? With whom do they engage in active play? | Bristol, England, UK. | Not specified. | Not specified. | Not specified. | 77 children aged 10-11 years. | Four high schools varying by SES selected for recruitment. 10-11 yr old children targeted. | Focus groups. | Thematic analysis. | Not specified. |
| Brockman, Jago, & Fox (2011) | Why do children engage in active play? What factors limit children's active play? What factors facilitate children's active play? | Bristol, England, UK. | Not specified. | Not specified. | Not specified. | 77 children aged 10-11 years (appears to be same sample as Brockman, Fox, & Jago 2011) | Four high schools varying by SES selected for recruitment. 10-11 yr old children targeted. | Focus groups | Thematic analysis | Themes discussed among second and third authors to check for consistency. |
| Burke (2005) | Explored primary school children's preferred spaces and places for play. | Leeds, England, UK. | Not specified. | Not specified. | Not specified. | 32 children aged 6-11 years | Two communities selected (one low SES). Children aged 7-11 targeted and recruited via schools. | Photo elicitation followed by interviews. | Not specified. | Not specified. |
| Dias & Whitaker (2013) | Black mothers’ perceptions of neighborhood safety and outdoor play | Newark, New Jersey, US. | Not specified. | Not specified. | Not specified. | 36 Black mothers with children aged 9-13 years residing in a low SES area. | Not specified. | Individual interviews and focus groups | Thematic analysis | Not specified. |
| Ferré et al. (2006) | Examined the ways in which playgrounds are constructed and experienced in children's daily lives. | Two medium sized cities in Spain. | Not specified. | Not specified. | Not specified. | Politicians, social service personnel and town planners and adult users. Sample size not specified. | Not specified. | In-depth individual interviews, informal interviews, 32 direct observations of playground areas. | Not specified. | Not specified. |
| Gearin & Kahle (2006) | Explored teen and adult perceptions of urban green space in Los Angeles. | Los Angeles, California, US. | Not specified. | Not specified. | Not specified. | 16 high school seniors (5 girls and 11 boys) and adults (number of adults not clear). | Not specified | Focus groups, survey, and an outdoor walk. | Not specified but results organized by themes. | Not specified. |
| Holt et al. (2015) | Examined factors that influenced childhood active free play. | Edmonton, Alberta, Canada. | Not specified. | Not specified. | Not specified. | 13 young adults (4 male, 9 female; average age = 19.31 years) from middle class families (n = 8) and lower middle class families (n = 5). | Purposeful sampling:  Participants must have lived in city for at least 10 years and be aged 18-21 years old | Individual interview and a walk-along interview. | Thematic analysis (Comprehending, synthesizing, theorizing, recontextualizing). | Information-rich participants, methods triangulation, two researchers completed analysis. |
| Jago et al. (2009) | Parental attitudes toward children’s independent physical activity. | Bristol, England, UK. | Not specified. | Not specified. | Not specified. | 24 parents (4 males) of 10-11 year old children. | Parents of 10-11 years olds sampled from low, middle, and high SES schools. | Telephone interviews. | Thematic analysis. | Codes checked by a second investigator. |
| Jansson (2010) | Explored factors affecting playground attractiveness from users' perspectives. | Glumslöv and Degeberga, Sweden. | Classification of features of attractive playgrounds created based on literature. | Not specified. | Not specified. | 86 children aged between 6 -11. Preschool teachers, preschool children's parents. | None specified. | Group interviews with children, survey, and direct observations of playgrounds. | Qualitative data analysis following the procedures described by Miles & Huberman, 1994. | Not specified. |
| Jenkins (2006) | Explored factors that can influence how parents and their adolescents and teenage offspring negotiate access to outdoor play. | South Wales, UK. | Not specified. | Not specified. | Not specified | 15 youth aged 11-15 years (11 male) and 13 mothers. | Purposeful sampling: Respondents recruited based on responses to a questionnaire completed during visits to an A&E ward | Semi-structured interviews. | Not specified | Not specified. |
| Karsten (2003) | Explored gender issues in children's outdoor play in Amsterdam | Amsterdam, The Netherlands. | Loftland’s (1985) classification of residents. | Broadly approached from a gender relations perspective. | Ethnographic. | Specific sample detailed not reported. | Eight playgrounds selected based on social and ethnic context. | Observation and interviews during the observations. | Not specified | Not specified, but methods triangulation possible. |
| Karsten (2005) | Compared children’s use of public spaces in 1950s and 1960s to that of the 2000s | Amsterdam, The Netherlands. | Not specified. | Not specified | Oral history. | 34 children, 22 parents, 16 ‘former children,’ 13 older neighbors, 14 professionals | Selection of individuals from three specific neighborhoods | Archives, interviews, observations | Profiles of three ‘types’ of children created | Not specified, but methods triangulation possible. |
| Kinoshita (2009) | Examined the transformation of children’s playing in a neighborhood | Tokyo, Japan | Not specified. | Not specified | Action research. | 75 individuals from ‘four generational groups’ (ages not specified) | Selection of individuals based on generation and residence in neighborhood | Interviews and creation of ‘play maps’ | Thematic analysis. Specific details not provided. | Not specified, but methods triangulation possible. |
| Kruger & Chawla (2005) | Explored children’s use of their local environments, the problems they face, and their own priorities for making Johannesburg a better city in which to grow up | Johannesburgh, South Africa. | Not specified. | Not specified. | Not specified. | Children within the four locations who met age criteria (10 through 14 years old). No sample size given. | Four locations selected based on differing geographic and socioeconomic areas. Participants were randomly selected from a list of potential participants | Children's drawings; discussion with children. | Not specifically mentioned. Some results were categorized by theme. | Not specified. |
| L'Aoustet & Griffet (2004) | To understand how the sharing of public spaces shapes youth socialization. | Marseille, France. | Not specified. | Not specified. | Ethnographic. | Specified details of sample not reported. | Selection of a specific public space (Borely park) | Simple visits to the grounds, observations, tape-recorded discussions with park users | Not specified. | Not specified. |
| Lee et al. (2013) | Examined children’s perceptions of fun, barriers, and facilitators affecting active free play. | Tokorozawa, Japan. | Not specified. | Not specified. | Not specified. | 60 children aged 9-11 years. | Children recruited from one school. Reasons for selection of school not specified. | Focus groups. | Thematic analysis. | Inter-rater cross-coding check of analysis. |
| Little (2013) | Examined mother’s beliefs about children’s outdoor play opportunities and exposure to, and management of, risks. | Sydney, Australia. | Framework of risk-taking used. | Not specified | Not specified | 26 mothers of 4-5 year old children | Purposeful sampling: Based on age of children and participants recruited from four sites representing different levels of SES. | Semi-structured individual interviews | Thematic analysis, constant comparison | Not specified. |
| Lloyd et al. (2008) | Examined the role of local neighborhood parks in the lives of teenage girls. | Brisbane, Australia. | Framed in adolescent development literature. | Social construction theory (Sarbin & Kituse, 1994). | Not specified. | 11 females aged 14 to 18 years | Purposeful sampling. Participants drawn from individuals to had been in one of three focus groups. Selected based on gender, age, place of residence, and use of park. | Semi-structured interviews. | Open, axial, and selective coding. | Not specified. |
| MacDougall et al. (2009) | Compared urban and rural children’s accounts of what area is like for them. Examined rules, boundaries, and who sets them. | Urban area (Adelaide) and rural area (Kangaroo Island), Australia | Not specified. | Not specified. | Not specified. | 33 children aged 8-10 years old. | Urban and rural areas selected. Voluntary participation from children recruited from two schools. | Focus groups, mapping, and photovoice, | Thematic analysis. Details of analysis not provided. | Not specified, but methods triangulation possible. |
| Matthews et al. (2000) | Explored ways in which children encounter the countryside and examine the 'rural' from the viewpoint of young people. | Northamptonshire, England, UK. | Used concept of a ‘fourth environment’ which is a rural geography outdoors (Matthews & Limb, 1999). | Not specified. | Not specified. | 372 individuals aged 9-16 years old completed questionnaires. 22 individuals aged 9-16 years old participated in focus groups interviews. Number of participants in ‘on the street’ interviews not reported. | Attempts made to stratify participants by gender and social class. | Questionnaire, semi-structured interviews ‘on the street’ plus three focus groups. | Three themes identified. Details of analysis not provided. | Not specified, but methods triangulation possible. |
| Maxey (1999) | Investigated the spatial and social places of play. | Wales, UK. | Not specified. | Not specified. | Not specified. | 30 participants (14 children or teenagers) for interviews. | Urban and rural sites selected. No sampling criteria for participants reported. | Participant observation and in-depth, unstructured interviews. | Not specified but results were organized by categories and themes. | Not specified, but methods triangulation possible. |
| Miller & Kuhaneck (2008) | Investigated perceptions of play experiences and rationale for play choices among children. experiences. | Not reported, likely US. | None a priori. Dynamic model for play choice presented as results. | Not specified. | Grounded theory. | 6 girls and 4 boys between the ages of 7 and 11 years. | Selection criteria based on age, cognitive ability, language skills, residence in varying environments, and number of siblings. | Semi-structured individual interviews. | Constant comparison method; open coding, axial coding, and selective coding. | ‘Triangulation of authors’ and peer debriefing. |
| Nicholson et al. (2014) | Examined children’s perspectives on children’s and adults’ play. | California, US. | Not specified. | Not specified. | Not specified. | 89 children (64 females, 34 males) aged 3-7 years. 37 African-America, 25 White, 17 Asian, 6 Latino/Hispanic, 5 Biracial, and 8 ethnicity/race not reported. | A priori sampling criteria not reported, but participants were drawn from a state school, private pre-schools, a child care program, and a final unspecified setting. | Semi-structured individual interviews. | Thematic analysis. | Inter-rater reliability coding check. |
| O'Brien & Smith (2002) | Explored how free outdoor play, away from adult supervision, has been affected by parents' fears about risk | Not clear, but most likely Brighton, England, UK. | Not specified. | Not specified. | Grounded theory. | 6 parents (aged 34-46 years) of 8 year old children. | Specific a priori sampling criteria not reported, other than report that a homogenous sample was chosen. | Individual interviews. | Thematic analysis, coding, categorization, axial coding. | Not specified. |
| Oke et al. (1999) | Situational analysis of play in an urban setting. To study the patterns of children's play in urban settings; to study the restrictions, hazards posed, as well as facilities offered by an urban environment; to study the manner in which children make creative adaptations of their environment | Urban settings in India | Not specified. | Not specified. | Ethnographic. | 240 children (130 boys and 100 girls) aged 6-12 years were observed. 72 children (36 male) and 5 teachers interviewed. | Settings selected based on SES and geographic features. ‘Instantaneous sampling’ and ‘activity sampling’ for observations. Sampling criteria for individual interviews not explained. | Participant and non-participant observations, interviews with children and adults. | Not specified. | Not specified, but methods triangulation possible. |
| Pinkster & Fortuijn (2009) | How neighbourhood effects are related to, and mediated by, other social domains in which children group up, specifically the family context. What degree do residents in a low income neighborhood regard their neighborhood as a problematic place to raise their children? To what degree are these negative influences moderated or mediated through parental strategies? | The Hague, The Netherlands. | Parenting strategies (Jarrett, 1997) | Not specified. | Case study. | 19 neighborhood ‘experts’ (officials, social workers, education works, law enforcement, residents, and volunteers). 46 residents in social hosing. | Members of three largest migrant groups (from Morocco, Turkey, and Suriname) recruited. | Individual semi-structured interviews. | Not specified but mentioned using a qualitative data-analysis software. Results a organized by themes. | Not specified. |
| Ries et al. (2008) | Investigated environmental factors influencing the use of recreational facilities for physical activity by urban African-American adolescents. | Baltimore, Maryland, US. | Not specified. | Not specified. | Grounded theory. | 48 African-American adolescents aged 14 to 18 years. | Criterion sampling: If individuals contacted by phone self-directed as African-American  Observations conducted at 24 public recreation facilities selected to represent range of areas of city. | Individual interviews, observations, documentary analysis. | Coding, categorization (following grounded theory). | Iterative process, triangulation (interviews and observations) saturation, member-checking. |
| Smith & Barker (2001) | Examined children's experiences of playing in the countryside and the impact of out-of-school care. | Rural areas in England and Wales, UK | Not specified. | Not specified. | Not specified. | 45 children, unspecified number of local policy-makers, childcare providers and play workers, and parents. | Not specified but purposeful selection of three research sites in rural areas to ensure sample represent socio-economic differences. | Interviews and questionnaire survey of parents. | Not specified. | Not specified. |
| Tandy (1999) | Evaluated the nature and location of children’s play, both at present and a generation ago. Further evaluated changes in children’s independent mobility over time. | Newcastle, New South Wales, Australia. | Not specified. | Not specified. | Not specified. | 421 children (between 5 – 12 years) and 165 parents (most between 31 to 40 years) | Not specified, other than selection of a particular suburban area. | Questionnaires, included open ended questions and children were asked to draw or write a story about “What I would like to do on a sunny day” (p. 156). | Not specified. Results were presented as quotes and drawings to support interpretation. | Not specified. |
| Tezel (2011) | Explored parents' concerns for their children’s use of outdoor space in a gated community. Parents were asked to describe their attitudes to allowing their children to use the residential quarter for play. | Istanbul, Turkey, | Ecological model used to guide development of interview guides. | Not specified. | Not specified. | 97 parents (62 from high SES, 35 from mid SES) who have at least one child in grade 1-8 | Gated community identified, then random selection of the community inhabitants and snowballing techniques. | Semi structured interviews. | Coded categories and sub categories. | Inter-rater cross-coding check of analysis. |
| Thomson & Philo (2004) | A deconstruction of play through an inquiry into the social geography of children’s play in a Scottish town. Explored what children think about their own play, what it entails and the spaces, places, social encounters, and social variations central to it. | Livingston, Scotland, UK. | Not specified, but concept of understanding geographies from children’s perspectives mentioned. Also used concepts of polymorphic and disordered spaces. | Not specified. | Not specified. | Three groups of children aged 8 and 9 years old from three different schools in Livingston. 73 participants in total. | All students within targeted age group from the schools participated. | Questionnaire with closed and open ended questions, mental map drawings, and focus group interviews. | Thematic analysis mentioned but not explained. | Not specified. |
| Tobin et al. (2013) | To examine how children's use of imagery in their active play can facilitate basic psychological needs. A secondary purpose was to examine the content of children's mental images associated with their active play. | London, Ontario, Canada. | Deci and Ryan's (2002) three basic psychological needs theory. | Not specified. | Not specified. | 48 boys and 56 girls aged 7-14 years old. | Not specified. | 23 focus groups. | Inductive and deductive thematic analysis using psychological needs theory. | Inter-rater reliability check on coding. |
| Tranter & Pawson (2001) | Examined the variability in children's independent access to local environments. | Christchurch, New Zealand. | Concept of child-friendly cities mentioned. | Not specified. | Case study. | Children aged between 9 to 11 (n = 436) and their parents (n = 297). Teachers and principals. | Schools selected to reflect variability in socio-economic status and traffic conditions near schools. Principals and 10 year old children then recruited | Questionnaires to parents and children; focus group interviews with children, (8-10 children per group); Individual interviews with principals, teachers, informal discussions with teachers and parents, observations. | Not specified. Qualitative results embedded in result section as direct quotes. | Not specified, but methods triangulation possible. |
| Tucker & Matthews (2001) | Explored girls' use of recreational spaces within rural areas. | Northamptonshire, England | Rural childhood ‘myth’ discussed. | Not specified. | Not specified. | Females aged 10-14 years old. Sample size not specified. | Voluntary participation. | In-depth discussions in groups of three or four girls. Semi-structured interviews; children's photographs; child-led video tour of the home settlement. | Not specified but appears to be thematic analysis. | Not specified, but methods triangulation likely. |
| Tucker et al. (2007) | Assessed parents’ preferences for neighbourhood parks. | London, Ontario, Canada. | Not specified. | Not specified. | Not specified. | 82 interviews with parents/guardians (56% mothers, 24% fathers, 7% grandparents, and 13% daycare providers) whose children were between the ages of 1-13 (85% being 7 or under) | Recruited voluntary participants ‘on the spot’ while they were watching their children at neighborhood parks | GIS mapping technique. Interviews, probes, and field notes. | Inductive content analysis of interviews and field notes. | Data saturation claimed. Member checking, inter-rater coding check. Addressed credibility, dependability, confirmability, and transferability. |
| Valentine [59] | Explored how an imagining of the countryside as an idealized place in which to grow up is constructed, mobilized, and contested by rural parents in their accounts of their children’s lives. | Rural village in Derbyshire, England, UK. | Myth of idyllic rural childhoods. | Not specified. | Not specified. | 10 ‘sets of parent(s)’ | Participants identified based on responses to larger questionnaire-based study. 5 ‘long term resident’ parents and 5 ‘incomers’ who had previously lived in towns or cities | Interviews. | Not specified. | Not specified. |
| Valentine (1997) | Children’s competence in relation to the ability of children to negotiate public spaces alone safely. | Metropolitan, non-metropolitan, and rural areas in Yorkshire, Cheshire, Derbyshire and Greater Manchester in England , UK | Hart’s concept of children’s spatial competence. | Not specified. | Ethnographic. | Children aged 8-11, their parents | Selected based on responses to questionnaire. Specific criteria not reported. | Semi-structured interviews, questionnaire, and ethnographic work, focus group discussions | Not specified, appears to be thematic analysis. | Not specified, but methods triangulation likely. |
| Valentine & McKendrick (1997) | Explored the extent to which parents consider that there are adequate public facilities and play opportunities in their neighborhoods for their children and whether children’s experiences of outdoor play are changing. | North-West England, UK. | Used concepts of geographies of children’s outdoor play. | Not specified. | Not specified. | Parents with a child aged between 8 and 11 years. 400 completed questionnaires and 70 completed interviews. | 70 households selected for interviews. Areas selected based on social class, child demography, and macro-geographical data. Specific criteria not reported. | Questionnaires and semi-structured interviews | ‘Conventional social science techniques for handling qualitative data” (p. 224). | Piloting of data collection techniques. |
| Veitch et al. (2006) | Investigated where children play and why, by exploring parents' perceptions of the individual, social and physical environment influences on their child's active free-play. | Melbourne, Australia. | Ecological model. | Not specified. | Not specified. | 78 parents (20 from high SES area; 35 from mid SES area; and 23 from low SES area). | Purposive sampling to ensure children from a wide range of SES backgrounds were represented. | Individual semi structured interviews. | Coding to identify categories, sub categories, then themes. | Random sample of 10 transcripts cross-checked for inter-coder reliability. |
| Veitch et al. (2007) | To examine children's view on the role and use of public open spaces for active free play. | Melbourne, Australia. | Ecological model. | Not specified. | Not specified. | 132 children (71 girls, 61 boys) aged 6-12 years from a selection of primary schools from metropolitan and out-urban Melbourne. 41 were from low SES area; 64 were from a mid SES area; and 28 from a high SES area. | Purposive sampling to ensure children from a range of SES backgrounds were represented. | Focus groups. | Thematic analysis guided by the ecological model. | Not specified. |
| Witten et al. (2013) | From parents' perspectives, what are the influences upon the intergenerational decline in children's independent outdoor play and active travel | Auckland, New Zealand. | Not specified. | Not specified. | Mixed methods. | 68 parents from the *Kids in the City* project. No further details provided. | Recruitment from six schools representing different income and walkability levels. | Focus groups. | Thematic analysis. No details provided. | Two authors read and discussed transcripts before creating coding framework. |
| Wridt (2004) | Social and spatial evolution of play and recreation in New York City from the 1930s to 2000s | New York City, US. | Not specified. | Not specified. | Ethnographic. | 3 participants (aged 60, 31, and 13 years old respectively). | Not reported. | Archival materials, fieldnotes, and autobiographies of participants (interviews, focus groups, mapping, walkalongs) | Individual profiles created. Specific analytic strategies not reported. | Not specified. |
