# Supplementary material for: A meta-study of qualitative research examining determinants of children’s independent active free play
Source: Int J Behav Nutr Phys Act. 2015 Jan 24;12:5. doi: 10.1186/s12966-015-0165-9 (PMC4318368; doi:10.1186/s12966-015-0165-9)
Supplement: Additional file 3: Table S2. — Factors That Influence Children’s Engagement in Active Free Play. [file 12966_2015_165_MOESM3_ESM.docx]

Additional file 3 Table S2 **Factors That Influence Children’s Engagement in Active Free Play**

| **Category** | **Theme** | **Studies** |
| --- | --- | --- |
| Child Characteristics |  |  |
|  | Age | [41,45-47,49,65,74-76] |
|  | Competence (‘streetwise’) | [49,55,63]. |
|  | Gender | [35,38,41,44-46,54,55,60,69,74] |
| Parental Restrictions |  |  |
|  | Safety: Strangers, bullies/teenagers, traffic  (Increased over time)  (Still a concern in rural areas) | [17,34,38,40,43,44,46-50,53,55,57-60,63,65,66,70,76]  [43,48,49,53,55,63,68,71]  [40,50,52-54] |
|  | Surveillance | [34,40,43,44,46-48,50,52,53,55,57,58,70] |
| Neighbourhood and Physical Environment |  |  |
|  | Fewer children in neighbourhood (Reduced ‘safety in numbers’) | [41,42,45,47,55,65,66,68,72,76] |
|  | Differences in preferences for play spaces between adults and children | [33-37,42-44,47,51,57,59,60,64,67,73-76] |
|  | Accessibility and proximity | [37,39,47,54,60,74,76] |
|  | Maintenance | [50,54,59,60,65,68,74] |
| Societal Changes |  |  |
|  | Reduced sense of community  (Need to revive community) | [42,46,50,53,55,60,62,63,66,72,76]  [53,57,63,66,72] |
|  | Good parenting ideal | [17,39,46,55,58,63,70] |
|  | Changing roles of parents | [62,68,71,72] |
|  | Privatisation of play spaces and playtime | [17,43,52,61,65,72]  [44,50,52-54,57,61] |
| Policy Issues |  |  |
|  | Give children voice | [33,38,40,44,46,50,54,55,59,74] |
